# Efficiency of Virucidal Disinfectants on Wood Surfaces in Animal Husbandry

**DOI:** 10.3390/microorganisms12051019

**Published:** 2024-05-17

**Authors:** Martin J. Oettler, Franz J. Conraths, Uwe Roesler, Sven Reiche, Timo Homeier-Bachmann, Nicolai Denzin

**Affiliations:** 1 Institute of Epidemiology, Friedrich-Loeffler-Institut, Federal Research Institute for Animal Health, 17493 Greifswald-Insel Riems, Germany; martin.oettler@fli.de (M.J.O.); franz.conraths@fli.de (F.J.C.); timo.homeier@fli.de (T.H.-B.); 2 Institute for Animal Hygiene and Environmental Health, Freie Universität Berlin, 14163 Berlin, Germany; uwe.roesler@fu-berlin.de; 3 Department of Experimental Animal Facilities and Biorisk Management, Friedrich-Loeffler-Institut, Federal Research Institute for Animal Health, 17493 Greifswald-Insel Riems, Germany; sven.reiche@fli.de

**Keywords:** disinfection, inactivation, wood, peracetic acid, formic acid, glutaraldehyde, Enterovirus E, Newcastle disease virus

## Abstract

The aim of this study was to test the inactivation of viruses on germ carriers of different types of wood using a disinfectant in order to assess the biosafety of wood as a building material in animal husbandry. The laboratory disinfectant efficacy tests were based on German testing guidelines and current European standards. Five different types of wood germ carriers, i.e., spruce (*Picea abies*), pine (*Pinus sylvestris*), poplar (*Populus* sp.), beech (*Fagus sylvatica*) and Douglas fir (*Pseudotsuga menziesii*), were inoculated with enveloped or non-enveloped viruses and then treated with one of three different disinfectants. The results revealed that intact, fine-sawn timber with a low roughness depth can be effectively inactivated. Peracetic acid proved to be the most effective disinfectant across all tests. Regardless of the pathogen and the type of wood, a concentration of 0.1% of the pure substance at a temperature of 10 °C and an exposure time of one hour can be recommended. At a temperature of −10 °C, a concentration of 0.75% is recommended. The basic chemicals formic acid and glutaraldehyde demonstrated only limited effectiveness overall. The synergistic effects of various wood components on the inactivation of viruses offer potential for further investigation. Disinfectant tests should also be conclusively verified in field trials to ensure that the results from standardised laboratory tests can be transferred to real stable conditions.

## 1. Introduction

The German Federal Ministry of Food and Agriculture recommends that wood as a natural building material should be given greater consideration in the construction of farm buildings [1]. Contrary to this recommendation, however, the importance of wood as an agricultural construction material in Germany has declined in recent years [2,3]. An argument in favour of the use of wood in agricultural construction is that the majority of farms also own woodland in addition to arable land and grassland [4,5,6]. In Germany, there are approximately 141,000 agricultural enterprises that also own forests with a total of approximately 15,500 km^2^ of woodland or short rotation plantations [4]. This self-owned wood can be effectively used for the construction of farm buildings [4,7,8,9,10,11].

Cleaning and disinfection play a central role in animal husbandry, both in general infection prevention and in the eradication of animal diseases [12,13]. In this context, wooden surfaces are considered to be materials that are difficult to hygienise [14]. However, there are studies from the food sector that attribute good hygienisation properties to wooden surfaces [15,16]. Due to the porous surface, the procedure of cleaning and disinfecting wooden surfaces in agriculture should be carried out with additional steps and details [17,18,19,20].

To ensure the effectiveness of the disinfectants used, various disinfectant testing guidelines have been established for the veterinary sector and specifically for the livestock sector. These differ in terms of the spectrum of pathogens and the surface characteristics to which the disinfectants are applied. In the field of animal husbandry, the germ carrier test on porous surfaces is considered to be the most important efficacy test [17]. For the testing of virucidal disinfectants in animal husbandry, a test method in suspension [21] and a test method on non-porous germ carriers [22] currently exist at the European level. In addition, there are also German national guidelines that cover the area of virucidal carrier testing for animal hygiene [23]. Based on these guidelines, several studies have already been carried out to optimise the disinfectant test procedure [24,25,26,27,28].

The use of wood as a traditional construction material in agriculture can contribute to a more sustainable economy, climate protection, and the strengthening of regional value chains. However, the aspects of animal hygiene and animal disease control must not be neglected. The research carried out and presented in this paper aims to assess the compatibility of the aforementioned requirements.

## 2. Materials and Methods

### 2.1. Cells and Viruses

Cell culture and virus propagation procedures were based on current European standards [29].

In brief, Madin–Darby bovine kidney (MDBK, CCLV-0261) cells and Leghorn male chicken hepatocellular carcinoma (LMH, CCLV-0417) cells were cultured using the appropriate culture medium—ZB5 (Appendix A Table A1) for MDBK and ZB9h (Appendix A Table A2) for LMH; both were mixed with 10% foetal calf serum (FCS). Cell cultures were incubated at 37 °C and 5% CO_2_. Both cell lines were obtained from the Bio Bank (Department of Experimental Animal Facilities and Biorisk Management) of the Friedrich-Loeffler-Institut, Federal Research Institute for Animal Health, Germany.

The test viruses used were the standard test viruses listed in the methods outlined by the German Veterinary Medical Society (Deutsche Veterinärmedizinische Gesellschaft, DVG) for testing chemical disinfectants in animal husbandry [23]. Enterovirus E (EV-E, strain LCR 4) was used as a non-enveloped virus, and Newcastle disease virus (NDV, strain Montana) was used as an enveloped virus. For EV-E, MDBK cells were used for propagation and as a detection system, and LMH cells were used for NDV. After virus propagation, the viruses were aliquoted into cryotubes and stored as a virus test suspension at −80 °C until use. Both virus strains were provided by the Institute for Animal Hygiene and Environmental Health of the Freie Universität Berlin, Germany.

### 2.2. Disinfectants

To assure comparability, biocidal basic chemicals rather than composed commercial products were employed as “disinfectants”. Peracetic acid (15%, AppliChem GmbH, Darmstadt, Germany), formic acid (≥98%, Carl Roth GmbH + Co. KG, Karlsruhe, Germany), and glutaraldehyde (50%, Sigma-Aldrich Chemie GmbH, Steinheim, Germany) were used. The disinfectants were selected with respect to their differing mechanisms of action (peroxide, organic acid, and aldehyde) and their relevance as disinfectants in agriculture [17,30,31,32].

All of these pure substances comply with the requirements of the EU Biocidal Products Regulation [33].

### 2.3. Germ Carriers

**Figure 1:**
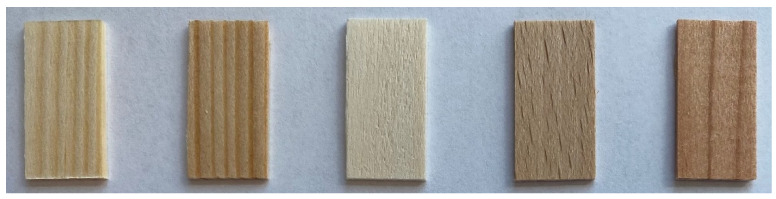
Germ carriers (from left to right: spruce, pine, poplar, beech, and Douglas fir).

In laboratory tests, porous materials were analysed as germ carriers. Wood from five different tree species was used (Figure 1). Spruce (*Picea abies*) and pine (*Pinus sylvestris*) discs were used as these are two classic German wood species used in construction. Poplar (*Populus* sp.) discs were analysed because this is the German [23] and European [34] standard for testing disinfectants on porous materials in animal husbandry. Beech (*Fagus sylvatica*) and Douglas fir (*Pseudotsuga menziesii*) discs were included in the test as representatives of modern construction woods.

All wooden germ carriers were cut from dried boards and sawn to a size of 20 mm × 10 mm and a thickness of 1 mm prior to testing. The surface was classified as fine-sawn and thus had a uniform, intact surface with a low roughness depth [35]. The wood discs were prepared and kindly provided by the Chair of Wood Science (School of Life Sciences) of the Technical University of Munich, Germany.

### 2.4. Performing the Inactivation Tests

The laboratory inactivation tests were based on the German DVG test guideline [23] in combination with current European standards [22,34].

**Figure 2:**
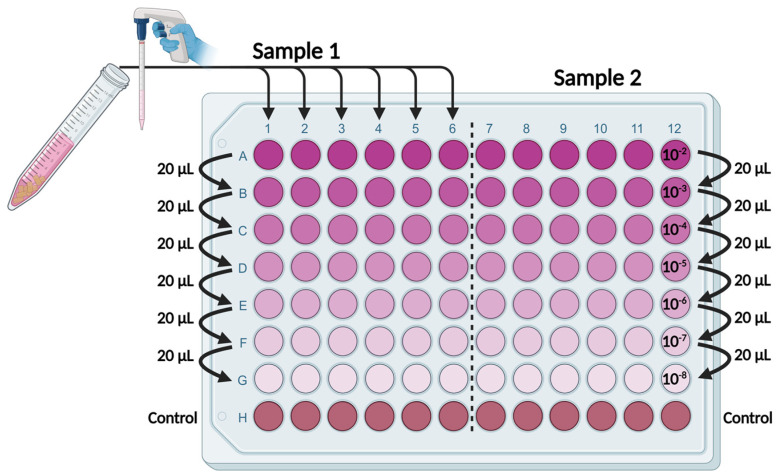
Titration scheme on a 96-well plate, created with Biorender.com.

Prior to the inactivation tests, the germ carriers were sterilized through autoclaving (121 °C, 2.8 MPa for 1.5 h) and then dried. For inoculation, the virus test suspension from the virus propagation was mixed with 3 g/L bovine serum albumin (Sigma-Aldrich Chemie GmbH, Steinheim, Germany) as a substance potentially interfering with the inactivation process. Each germ carrier was inoculated with 100 µL of this mixture and then completely dried in a silica gel desiccator under vacuum (approximately 80 kPa negative pressure) and at room temperature. For inactivation, the germ carriers were transferred to a 6-well plate and treated with 4 mL of a biocidal basic chemical. Each well was filled with a different concentration. The respective concentrations of biocidal basic chemicals were freshly prepared with hard water (composition described in Appendix A Table A3) *v*/*v* on the day of the test. As a control, one well of the 6-well plate was filled with 4 mL of hard water only. The respective germ carrier, without contact with a diluted basic chemical, was used to establish the reference titre of the test. Subsequently, the plate was incubated for one hour at 10 °C (or −10 °C). Then, the germ carriers were removed and transferred to 9.9 mL of the respective cell culture medium (containing 2% FCS) for neutralisation. The germ carriers were cut into eight pieces using sterile scissors. To desorb the viruses, the solutions were vortexed, treated in an ice-water-filled ultrasonic bath for 10 min, and then centrifuged at 1600× *g_N_* for 15 min. Thus, the resulting virus dilution was 10^−2^ relative to the initial inoculum. The titration of the samples was carried out in 96-well plates (Figure 2).

The wells of rows B-H were prefilled with 180 µL of the respective cell culture medium (containing 2% FCS). At least 120 µL of the virus sample suspension was transferred to row A; then, 20 µL was pipetted into the next dilution row and mixed, and the process was repeated down to row G. Row H served as a negative control without the addition of virus dilutions. For the incubation of a titration sample, 100 µL per well was transferred to a 96-well cell culture plate and incubated at 37 °C and 5% CO_2_. If cytotoxicity or a low virus load was encountered, the protocol had to be adapted, and 100 µL or 1 mL, respectively, was transferred to a 12-well cell culture plate instead and then incubated.

The evaluation was based on the presence or absence of virus-specific cytopathic effects, assessed via inverse microscopy, and virus titres were calculated using the Spearman–Kärber formula (Appendix A Equation (A1)) [36,37]. In order to calculate the virus reduction in the respective concentration of the biocidal basic chemical, the test titre was subtracted from the reference titre (germ carrier in hard water). The test was considered successful if the transition from non-effective inactivation to effective inactivation could be demonstrated by the different concentration gradations chosen for the biocidal basic chemical; otherwise, the concentration was adjusted accordingly to capture the aforementioned transition. Effective inactivation was defined as a reduction in the virus concentration of at least four decadal logarithmic levels [23]. Each test was repeated twice; i.e., three separate experiments were conducted. The highest minimum concentration of the chemical required for effective inactivation in three independent consecutive tests was conservatively defined as the final effective concentration of the chemical.

### 2.5. Statistics

R version 4.3.1 software for Windows [38] was used to test for statistically significant differences. For a statistical comparison of the results, a conservative assumption was made in that the values were not normally distributed as the number of data points was small. Accordingly, non-parametric tests were used. The Mann–Whitney U test was used to analyse the effective concentrations of the inactivation tests when comparing only two groups, and the Kruskal–Wallis test was used when comparing more than two groups. A *p*-value of 0.05 was defined as the significance level for differences to be detected.

## 3. Results

### 3.1. Germ Carrier Tests with Peracetic Acid at 10 °C

Peracetic acid was found to be efficient against both viral pathogens and effective inactivation was possible on all wood types tested. EV-E was more resistant to the sterilant than NDV, as evidenced by the higher concentrations of peracetic acid required. Regardless of the type of wood, there was a statistically significant difference in the effective concentrations between the two viruses (*p* < 0.0001).

For EV-E as non-enveloped viruses, concentrations of peracetic acid ranging from 0.025% to 0.1% were required for the effective inactivation of four log levels on the wood surfaces (Table 1). In terms of the highest effective concentration of each of the three replicates (final concentration), which was conservatively considered to be the appropriate result of the test design, pine and poplar—each at 0.05% peroxide—were the woods requiring the lowest concentrations. The other woods were just above these values with 0.075% for spruce and beech and 0.1% for Douglas fir. The concentrations determined for the different wood species were not significantly different (*p* = 0.10).

For NDV as an enveloped virus, a range of concentrations from 0.001% to 0.025% peroxide was required for effective inactivation (Table 2). Pine was found to be the wood species requiring the lowest concentration of pure substance for effective inactivation, with a final concentration of 0.0025% peracetic acid. The other woods required higher final concentrations, with 0.01% for spruce and 0.025% for poplar, beech, and Douglas fir. There were significant differences (*p* < 0.05) between the wood species in the concentrations determined for the enveloped virus.

### 3.2. Germ Carrier Tests with Formic Acid at 10 °C

Formic acid was also found to be effective against both viruses and all types of wood tested. Differences in the susceptibility of the two viruses relative to formic acid were generally small. For spruce, pine and poplar, EV-E was the virus that required higher concentrations. For beech, this was the case for NDV. In Douglas fir, the concentrations for both viruses were similar. Regardless of the wood species, there was no statistically significant difference (*p* = 0.07) in the effective concentrations between the two viruses.

For the non-enveloped EV-E, the required concentrations for a reduction of at least four decadal log levels on the different woods ranged from 0.75% to 1.5% of the organic acid (Table 3). Douglas fir turned out to be the wood requiring the lowest final concentration of formic acid (0.75%) for an effective inactivation. Values for pine and beech were slightly higher at 1.0%, as were spruce and poplar (1.5%). There were no significant differences between the wood species regarding the concentrations required for the effective inactivation of EV-E (*p* = 0.055).

For the enveloped NDV, a concentration range of 0.25% to 2.0% of the basic chemical was found to be necessary for effective inactivation (Table 4). In general, coniferous woods require lower concentrations of formic acid than deciduous woods. The best or lowest result of the three tests carried out (final concentration) was obtained on pine with a concentration of 0.25% formic acid. The other conifers required 0.5% of the basic chemical on spruce and 0.75% on Douglas fir. For effective inactivation, the deciduous woods required a concentration of 1.0% of the pure substance for poplar and 2.0% for beech. There was a statistically significant difference (*p* < 0.05) between the concentrations required by different woods in the inactivation tests.

### 3.3. Germ Carrier Tests with Glutaraldehyde at 10 °C

In contrast to the other two basic chemicals, glutaraldehyde showed no general efficacy. In the tests carried out, the criteria for effective inactivation were only met for the enveloped virus NDV but not for the non-enveloped virus EV-E.

Only the limited efficacy of glutaraldehyde was observed for EV-E. A reduction in pathogens of four decadal logarithms was not observed in any of the tests carried out (Table 5). Concentrations ranging from 1.0% to 10.0% of the pure substance were analysed. Depending on the type of wood tested, usually, a reduction of only two to three decadal logarithmic levels compared to the reference titre was demonstrable even at the maximum concentration tested (10.0%). The titre reductions on the different wood species by 10.0% glutaraldehyde were not significantly different (*p* = 0.14).

For the effective inactivation of NDV, concentrations of aldehyde ranging from 0.075% to 1.5% were found for different types of wood (Table 6). As with formic acid, lower concentrations were required for effective inactivation on coniferous wood than on deciduous wood. Glutaraldehyde was found to be effective on pine at a very low final concentration of 0.1%. The other two conifers tested required 0.75% of aldehyde, and the two deciduous woods required 1.5%. There were significant differences in the concentrations determined for the enveloped virus between the different types of wood (*p* < 0.05).

### 3.4. Germ Carrier Tests at −10 °C

Inactivation tests at −10 °C were only carried out for EV-E, as this virus proved to be the limiting virus in the tests at 10 °C among the viruses used. This was demonstrated by the higher or equal final concentrations of the basic chemicals required for effective inactivation (with the exception of formic acid on beech wood).

In the case of peracetic acid, the concentrations of peroxide required for effective inactivation ranged from 0.25% to 0.75% (Table 7). The lowest concentrations for final effective inactivation were found for spruce and poplar wood at 0.25% of the pure substance. This was followed by pine and Douglas fir at 0.5% each and beech at 0.75%. There were significant differences (*p* < 0.05) between the concentrations obtained for effective inactivation on the different wood species. Across all tested woods, the concentrations of peracetic acid required for effective inactivation at −10 °C were significantly higher (*p* < 0.0001) compared to 10 °C.

For formic acid, a concentration range of 2.5% to 5.0% of the organic acid was found to be necessary for effective inactivation (Table 8). Spruce, poplar, and beech were found to require the lowest concentrations of disinfectant, with a final concentration of 3.0% of the pure substance. The other woods followed with 4.0% for Douglas fir and 5.0% for pine. Results were significantly different (*p* < 0.05) between the wood species under test. Across all woods, the concentrations of formic acid required at −10 °C were significantly higher (*p* < 0.0001) compared to 10 °C.

Glutaraldehyde was not tested for inactivation at −10 °C. Based on the lack of efficacy of the aldehyde at 10 °C against the non-enveloped virus and the known poor efficacy of glutaraldehyde at cold temperatures (see below), it was assumed that there was no sufficient efficacy at −10 °C.

### 3.5. Detailed Results

The full results can be found in the Supplementary Materials (Tables S1–S40).

## 4. Discussion

### 4.1. Inactivation Tests with Non-Enveloped Viruses

Enterovirus E proved very suitable for carrying out inactivation tests with non-enveloped viruses. The virus could be propagated in cell culture to high titres and was easily detected by a specific CPE. This very good suitability of EV-E as a test virus has also been confirmed in other studies [24,25,26,39]. It is also highly relevant as a test virus, as it is one of the two non-enveloped test viruses in the DVG test guidelines [23] and is also established as a test virus in a European standard [21].

With regard to peracetic acid, concentrations of 0.05% to 0.1% were found to be effective on different types of wood. Pirschel [25] reported a similar result (0.05% with 1 h incubation) in a study on poplar wood also using the DVG method. Studies employing suspension tests [24,39,40] yielded comparable results with respect to the required concentration of peracetic acid and exposure time. Other authors [41,42] mention a concentration of 0.2% peracetic acid but with an exposure time of only several minutes.

Concerning formic acid, a range of concentrations from 0.75% to 1.5% was found to be effective on the different types of wood. Several studies [25,41,43,44] indicate a similar concentration range of 0.5% to 2% for the germ carrier test on poplar wood. In suspension tests, the required concentration or exposure time was halved [24,39,41,43,44].

The basic chemical glutaraldehyde turned out to be unsuitable for the effective inactivation of the non-enveloped EV-E. Concentrations of up to 10% were tested, which is well above the practical standard. Pirschel [25] also conducted germ carrier tests on poplar wood and found that 2.0% glutaraldehyde (and an exposure time of 2 h) did not even produce a reproducible reduction in titre over three decadal logarithms. This lack of efficacy was also demonstrated in suspension tests by Rhee et al. [45]. Mahnel [46] also described a high resistance of enteroviruses to aldehydes. On the basis of the tests carried out and the results from the literature, it can be assumed that the lack of efficacy of glutaraldehyde against EV-E is not related to the surface being inactivated. In this case, it is not the type of wood that is responsible, but the basic chemical is generally insufficiently effective against this virus.

### 4.2. Inactivation Tests with Enveloped Viruses

The Newcastle disease virus proved to be suitable for inactivation experiments with enveloped viruses. The virus was able to multiply to high titres in permanent cell culture and was easily detected via the specific CPE.

NDV is relevant as a test virus because it is one of the two enveloped test viruses in the DVG test guidelines [23]. Its suitability as a test virus has been confirmed in other studies [26,27,39].

For peracetic acid, concentrations of 0.0025% to 0.025% were considered to be effective on the different types of wood, although significant differences were found. Schmidt [27] found similar results in germ carrier tests on poplar wood, although effective inactivation was defined only as a virus reduction of three decadal logarithmic levels. Comparable results were obtained in suspension tests by Al-Khleif [39]. According to Kramer et al. [42], 0.2% peracetic acid is required to achieve the inactivation of NDV within a few seconds.

In the trial with formic acid, significantly different concentrations ranging from 0.25% to 2% were found to be effective on wood samples. Similar results were obtained in suspension tests with the same virus–disinfectant combination by Al-Khleif [39]. According to Rheinbaben and Wolff [41], formic acid inactivates NDV within 5 min at a concentration of 2% or 15 min at 1%, respectively.

Regarding glutaraldehyde, significantly different concentrations of 0.1% to 1.5% were found to be effective in the woods tested. In a test on poplar wood, Schmidt [27] reported a result of 0.25%, where the aim of effective inactivation was only to reduce germs by three decadal logarithmic levels. The markedly lower concentrations in poplar wood in the latter study compared to the present one may be attributed to the lower threshold set for effective inactivation and the higher test temperature (20 °C), as glutaraldehyde is poorly effective at cold temperatures [17,47]. Rhee et al. [48] also found similar results in suspension tests.

### 4.3. Inactivation Tests at −10 °C

Inactivation tests at −10 °C were only carried out with EV-E, as this virus generally required higher concentrations of disinfectant than NDV at 10 °C and was therefore considered to be limiting.

The best results were obtained with peracetic acid. In the tests at −10 °C, concentrations of only 0.25% to 0.75% were found to be effective on wood species. This approximately corresponds to the usual commercial concentrations. The good performance is due to the excellent cold tolerance of peroxide [49]. According to Jones et al. [50], peracetic acid can be used at temperatures as low as −40 °C with the addition of an antifreeze.

Formic acid also demonstrated effective inactivation at −10 °C at concentrations of 3% to 5% on the effective wood species. These rather high concentrations can be explained by the limited effectiveness of the organic acid at cold temperatures [51]. According to Hölzle et al. [52], organic acids cannot be used as the sole active ingredient of disinfectants at temperatures below 10 °C.

In summary, the presented results of the inactivation tests at −10 °C are in line with the findings of Schliesser [49]. The author describes that the best disinfection results were achieved with peracetic acid at temperatures below the freezing point. Organic acids (including formic acid) were still found to be effective, although less than peroxide. The worst results were described for formaldehyde as a representative of the aldehydes, with an effect that took days to develop. Bremer [24] also carried out disinfectant tests, taking into account the test temperature, but only down to a temperature of 4 °C with an order of effectiveness with respect to the pure substances matching that of Schliesser [49] and the presented study.

### 4.4. General Performance of Disinfectants

Peracetic acid proved to be the most effective disinfectant in this study. Effective inactivation was achieved on all surfaces tested. In addition, as also described by Kramer [53], peroxide was characterised by a fast mode of action and a comparatively low influence with respect to ambient parameters. According to Schubert [30], peracetic acid has been used for many years in agriculture for the disinfection of surfaces, objects, and equipment. Ticháček [31] also recommends peracetic acid for use on large surfaces or in large-scale agricultural operations. Kramer et al. [42] classify peracetic acid as the ideal microbicidal and virucidal agent. With regard to practical disinfection, Böhm [17] recommends concentrations between 0.1% and 1% of the active substance for surface disinfection in agriculture, but more detailed information on the pathogen spectrum was not provided. Kramer et al. [42] mention the use of 0.2% peracetic acid as a sterilant in animal husbandry. Schliesser [49] also proposes similar basic chemical values of 0.2% to 0.4% for use in disinfection. Ticháček [31] mentions a pure substance concentration range of 0.01% to 2% for disinfecting objects made of wood, plastic, or metal. In the case of animal disease peracetic acid, a concentration of 0.4% is recommended as a surface sterilant with a minimum contact time of one hour [54].

Formic acid was also found to be an effective disinfectant in this study. Effective inactivation was achieved on all surfaces tested. The level set for statistical significance was narrowly missed in the comparison of the results of the tested viruses, which may be due to the limited number of samples used, i.e., the results might have become statistically significant if more replicates had been tested in parallel. However, the concentrations required at −10 °C were relatively high. This confirms the low efficacy of organic acids at low temperatures reported by Böhm [17] and Lächele [51]. Böhm [17] describes the use of formic acid mainly in stables, especially for surface disinfection. According to him, organic acid is used in concentrations of 2% to 4%. Kramer et al. [32] also describe the use of the basic chemical in the disinfection of livestock buildings. Depending on the pathogen, concentrations of 1% to 4% with a contact time of two hours are mentioned. The use of 4% formic acid with a minimum contact time of two hours is recommended for surface disinfection during animal disease epidemics [52].

Glutaraldehyde was only partially effective as a disinfectant in this study. Effective inactivation was achieved at 10 °C for the enveloped virus but not for the non-enveloped test virus. This was not tested at −10 °C; however, based on the poor efficacy at low temperatures reported by Michels et al. [47] and Böhm [17], it can be assumed that there would be no sufficient efficacy against the enveloped virus at −10 °C either. Kramer et al. [55] describe the virucidal effect of aldehyde at concentrations of 0.5% to 2% depending on the viruses to be inactivated. According to the authors, glutaraldehyde is used as a component in surface disinfectants, especially in combination with more aldehydes or other additives. The statement that glutaraldehyde can be used successfully in combination with other active ingredients is frequently found in the scientific literature [17,30,49,53,55,56]. In the event of an outbreak, the use of a glutaraldehyde solution is recommended for the inactivation of bacteria or bacterial spores. However, the disinfectant is not listed for the inactivation of animal epidemic viruses [47].

### 4.5. General Performance of Woods

It could be demonstrated that fine-sawn timber may be effectively inactivated depending on the temperature and type of disinfectant employed. Irrespective of the type of virus used, coniferous woods, especially pine, demonstrated the best hygienic properties with respect to surface disinfection. The different concentrations of basic chemicals needed on the tested woods are probably related to the specific content of wood constituents. Pine wood contains higher concentrations of constituents than the other woods tested at 9% compared to between 1.5% and 6% concerning the other woods [57]. In comparison to other porous building materials, such as concrete, wood is also a material that is easier to clean and disinfect [58].

In particular, the better-performing wood of the tested conifers is available in large quantities in German forests [59] and is also used effectively in the construction of farm buildings [5,7,9,10]. According to the presented laboratory findings, the use of wood as a construction material in animal husbandry is not compromised by the hygienic properties of standard timbers. Of course, further research should be carried out, particularly under field conditions, with respect to the poorer surface quality of wood. The potential synergistic effects of wood components with disinfection may deserve special attention in future studies.

## Figures and Tables

**Table 1:** Concentration of peracetic acid for the effective inactivation of EV-E at 10 °C in three tests.

	Spruce	Pine	Poplar	Beech	Douglas Fir
	0.075%	0.025%	0.05%	0.05%	0.05%
EV-E	0.075%	0.05%	0.05%	0.075%	0.05%
	0.075%	0.05%	0.05%	0.075%	0.1%

**Table 2:** Concentration of peracetic acid for the effective inactivation of NDV at 10 °C in three tests.

	Spruce	Pine	Poplar	Beech	Douglas Fir
	0.0075%	0.001%	0.025%	0.01%	0.0075%
NDV	0.01%	0.001%	0.025%	0.025%	0.01%
	0.01%	0.0025%	0.025%	0.025%	0.025%

**Table 3:** Concentration of formic acid for the effective inactivation of EV-E at 10 °C in three tests.

	Spruce	Pine	Poplar	Beech	Douglas Fir
	1.0%	0.75%	1.0%	0.75%	0.75%
EV-E	1.5%	1.0%	1.0%	1.0%	0.75%
	1.5%	1.0%	1.5%	1.0%	0.75%

**Table 4:** Concentration of formic acid for the effective inactivation of NDV at 10 °C in three tests.

	Spruce	Pine	Poplar	Beech	Douglas Fir
	0.25%	0.25%	1.0%	1.5%	0.25%
NDV	0.5%	0.25%	1.0%	1.5%	0.5%
	0.5%	0.25%	1.0%	2.0%	0.75%

**Table 5:** Titre reduction in log_10_TCID_50_/mL by 10% glutaraldehyde at 10 °C in three tests.

	Spruce	Pine	Poplar	Beech	Douglas Fir
	≥2.33	≥2.00	≥1.67	≥2.83	≥2.00
EV-E	≥2.83	≥2.83	≥1.83	≥2.83	≥2.50
	≥3.50	≥3.17	≥2.50	≥2.83	≥2.67

Further investigations indicated with high confidence that the reduction was less than 4 log_10_TCID_50_/mL in all tests.

**Table 6:** Concentration of glutaraldehyde for the effective inactivation of NDV at 10 °C in three tests.

	Spruce	Pine	Poplar	Beech	Douglas Fir
	0.5%	0.075%	1.5%	1.0%	0.5%
NDV	0.75%	0.075%	1.5%	1.5%	0.5%
	0.75%	0.1%	1.5%	1.5%	0.75%

**Table 7:** Concentration of peracetic acid for the effective inactivation of EV-E at −10 °C in three tests.

	Spruce	Pine	Poplar	Beech	Douglas Fir
	0.25%	0.5%	0.25%	0.5%	0.5%
EV-E	0.25%	0.5%	0.25%	0.5%	0.5%
	0.25%	0.5%	0.25%	0.75%	0.5%

**Table 8:** Concentration of formic acid for the effective inactivation of EV-E at −10 °C in three tests.

	Spruce	Pine	Poplar	Beech	Douglas Fir
	2.5%	5.0%	2.5%	2.5%	3.0%
EV-E	2.5%	5.0%	3.0%	2.5%	3.5%
	3.0%	5.0%	3.0%	3.0%	4.0%

## Data Availability

The original contributions presented in the study are included in the article and Supplementary Materials; further inquiries can be directed to the corresponding author.

## Supplementary Materials

The following supporting information can be downloaded at: https://www.mdpi.com/article/10.3390/microorganisms12051019/s1; Tables S1–S40.

## Appendix A

**Table A1:** Recipe for cell culture medium ZB5.

Component	Per Litre
MEM Eagle (Hanks’ Salts)	5.32 g
MEM (Earles’ Salts)	4.76 g
NaHCO_3_	1.25 g
Non-essential amino acids (NEA, 100x)	10 mL
Na-pyruvate	120 mg
pH 7.2	

**Table A2:** Recipe for cell culture medium ZB9h.

Component	Per Litre
DMEM	9.9 g
NaHCO_3_	860 mg
pH 7.2	

**Table A3:** Recipe for hard water.

	Component	Per Litre
	MgCl_2_	19.84 g
Solution A	CaCl_2_	46.24 g
	Membrane filtration	
Solution B	NaHCO_3_	35.02 g
	Membrane filtration	
Hard water	Solution A	6.0 mL
	Solution B	8.0 mL
	pH 7.0 ± 0.2	
	At −10 °C Ethylene glycol	25%

Equation (A1). Spearman–Kärber formula [36,37].

m = xk + d/2 − d Σ pi (A1)

m—The negative decadic logarithm of the titre based on the test volume in the wells, given in log TCID_50_/volume

x_k_—The logarithm value of the lowest dilution level at which all wells of a dilution series have a CPE

d—The logarithm value of the dilution factor

p_i_—The observed reaction rate (proportion of wells with CPE in a row)

Σ p_i_—The sum of the reaction rate from x_k_ to the highest dilution level
