# Supplementary material for: Efficiency of Virucidal Disinfectants on Wood Surfaces in Animal Husbandry
Source: Microorganisms. 2024 May 17;12(5):1019. doi: 10.3390/microorganisms12051019 (PMC11124466; doi:10.3390/microorganisms12051019)
Supplement: Supplementary file 1 [file microorganisms-12-01019-s001.zip › microorganisms-2957109-supplementary.pdf]

Table S1. EV-E on spruce wood with peracetic acid at 10 °C and 60 min exposure time.

Experiment	Concentration in % (v/v)	Titre in $log_{10}TCID_{50}/mL$	Reduction in $log_{10}TCID_{50}/mL$
1	Hard water	7.00	
	0.01	4.67	2.33
	0.025	3.83	3.17
	0.05	≤ 3.83	≥ 3.17
	0.075	≤ 2.50	≥ 4.50
	0.1	≤ 2.50	≥ 4.50
2	Hard water	7.00	
	0.01	5.00	2.00
	0.025	4.33	2.67
	0.05	≤ 3.50	≥ 3.50
	0.075	≤ 2.50	≥ 4.50
	0.1	≤ 2.50	≥ 4.50
3	Hard water	7.33	
	0.01	5.67	1.67
	0.025	5.17	2.17
	0.05	5.00	2.33
	0.075	≤ 2.67	≥ 4.67
	0.1	≤ 2.50	≥ 4.83

Table S2. EV-E on pine wood with peracetic acid at 10 °C and 60 min exposure time.

Experiment	Concentration in % (v/v)	Titre in $log_{10}TCID_{50}/mL$	Reduction in $log_{10}TCID_{50}/mL$
1	Hard water	7.17	
	0.0075	5.33	1.83
	0.01	5.67	1.50
	0.025	4.17	3.00
	0.05	≤ 2.50	≥ 4.67
	0.075	≤ 2.50	≥ 4.67
2	Hard water	7.33	
	0.0075	5.33	2.00
	0.01	3.67	3.67
	0.025	≤ 3.17	≥ 4.17
	0.05	≤ 2.50	≥ 4.83
	0.075	≤ 2.50	≥ 4.83
3	Hard water	7.00	
	0.0075	5.33	1.67
	0.01	4.83	2.17
	0.025	3.50	3.50
	0.05	≤ 3.00	≥ 4.00
	0.075	≤ 2.67	≥ 4.33

Table S3. EV-E on poplar wood with peracetic acid at 10 °C and 60 min exposure time.

Experiment	Concentration in % (v/v)	Titre in $log_{10}TCID_{50}/mL$	Reduction in $log_{10}TCID_{50}/mL$
1	Hard water	6.83	
	0.01	5.50	1.33
	0.025	≤ 3.00	≥ 3.83
	0.05	≤ 2.50	≥ 4.33
	0.075	≤ 2.50	≥ 4.33
	0.1	≤ 2.50	≥ 4.33
2	Hard water	6.83	
	0.01	4.33	2.50
	0.025	4.00	2.83
	0.05	≤ 2.50	≥ 4.33
	0.075	≤ 2.50	≥ 4.33
	0.1	≤ 2.50	≥ 4.33
3	Hard water	7.00	
	0.01	5.67	1.33
	0.025	≤ 3.50	≥ 3.50
	0.05	≤ 2.50	≥ 4.50
	0.075	≤ 2.50	≥ 4.50
	0.1	≤ 2.50	≥ 4.50

Table S4. EV-E on beech wood with peracetic acid at 10 °C and 60 min exposure time.

Experiment	Concentration in % (v/v)	Titre in $log_{10}TCID_{50}/mL$	Reduction in $log_{10}TCID_{50}/mL$
1	Hard water	7.50	
	0.01	5.33	2.17
	0.025	4.33	3.17
	0.05	4.67	2.83
	0.075	≤ 2.50	≥ 5.00
	0.1	≤ 2.50	≥ 5.00
2	Hard water	7.67	
	0.01	5.67	2.00
	0.025	4.50	3.17
	0.05	≤ 3.17	≥ 4.50
	0.075	≤ 2.67	≥ 5.00
	0.1	≤ 2.50	≥ 5.17
3	Hard water	7.33	
	0.01	5.67	1.67
	0.025	≤ 3.67	≥ 3.67
	0.05	≤ 3.67	≥ 3.67
	0.075	≤ 2.50	≥ 4.83
	0.1	≤ 2.50	≥ 4.83

Table S5. EV-E on Douglas fir wood with peracetic acid at 10 °C and 60 min exposure time.

Experiment	Concentration in % (v/v)	Titre in $log_{10}TCID_{50}/mL$	Reduction in $log_{10}TCID_{50}/mL$
1	Hard water	7.50	
	0.01	6.00	1.50
	0.025	5.17	2.33
	0.05	≤ 3.17	≥ 4.33
	0.075	≤ 2.83	≥ 4.67
	0.1	≤ 2.50	≥ 5.00
2	Hard water	6.83	
	0.01	6.00	0.83
	0.025	≤ 3.33	≥ 3.50
	0.05	4.00	2.83
	0.075	≤ 3.33	≥ 3.50
	0.1	≤ 2.50	≥ 4.33
3	Hard water	7.67	
	0.01	6.17	1.50
	0.025	5.33	2.33
	0.05	≤ 2.83	≥ 4.83
	0.075	≤ 3.33	≥ 4.33
	0.1	≤ 2.83	≥ 4.83

Table S6. NDV on spruce wood with peracetic acid at 10 °C and 60 min exposure time.

Experiment	Concentration in % (v/v)	Titre in $log_{10}TCID_{50}/mL$	Reduction in $log_{10}TCID_{50}/mL$
1	Hard water	7.67	
	0.0025	4.83	2.83
	0.005	4.50	3.17
	0.0075	4.67	3.00
	0.01	≤ 2.50	≥ 5.17
	0.025	≤ 2.50	≥ 5.17
2	Hard water	7.17	
	0.0025	5.00	2.17
	0.005	3.67	3.50
	0.0075	3.67	3.50
	0.01	≤ 2.67	≥ 4.50
	0.025	≤ 2.67	≥ 4.50
3	Hard water	6.50	
	0.0025	4.33	2.17
	0.005	≤ 3.50	≥ 3.00
	0.0075	≤ 2.50	≥ 4.00
	0.01	≤ 2.50	≥ 4.00
	0.025	≤ 2.50	≥ 4.00

Table S7. NDV on pine wood with peracetic acid at 10 °C and 60 min exposure time.

Experiment	Concentration in % (v/v)	Titre in $log_{10}TCID_{50}/mL$	Reduction in $log_{10}TCID_{50}/mL$
1	Hard water	6.00	
	0.00025	3.67	2.33
	0.0005	3.83	2.17
	0.00075	2.50	3.50
	0.001	≤ 1.67	≥ 4.33
	0.0025	≤ 1.50	≥ 4.50
2	Hard water	5.83	
	0.00025	3.83	2.00
	0.0005	3.17	2.67
	0.00075	2.83	3.00
	0.001	≤ 2.33	≥ 3.50
	0.0025	≤ 1.50	≥ 4.33
3	Hard water	5.83	
	0.00025	3.50	2.33
	0.0005	3.67	2.17
	0.00075	3.00	2.83
	0.001	≤ 1.50	≥ 4.33
	0.0025	≤ 1.50	≥ 4.33

Table S8. NDV on poplar wood with peracetic acid at 10 °C and 60 min exposure time.

Experiment	Concentration in % (v/v)	Titre in $log_{10}TCID_{50}/mL$	Reduction in $log_{10}TCID_{50}/mL$
1	Hard water	7.33	
	0.0025	5.33	2.00
	0.005	4.67	2.67
	0.0075	4.17	3.17
	0.01	≤ 4.00	≥ 3.33
	0.025	≤ 2.50	≥ 4.83
2	Hard water	6.50	
	0.0025	4.33	2.17
	0.005	4.00	2.50
	0.0075	3.83	2.67
	0.01	≤ 2.67	≥ 3.83
	0.025	≤ 2.50	≥ 4.00
3	Hard water	6.50	
	0.0025	≤ 3.67	≥ 2.83
	0.005	≤ 3.50	≥ 3.00
	0.0075	3.67	2.83
	0.01	≤ 2.83	≥ 3.67
	0.025	≤ 2.50	≥ 4.00

Table S9. NDV on beech wood with peracetic acid at 10 °C and 60 min exposure time.

Experiment	Concentration in % (v/v)	Titre in $log_{10}TCID_{50}/mL$	Reduction in $log_{10}TCID_{50}/mL$
1	Hard water	7.17	
	0.0025	4.83	2.33
	0.005	4.83	2.33
	0.0075	5.67	1.50
	0.01	3.50	3.67
	0.025	≤ 2.50	≥ 4.67
2	Hard water	7.83	
	0.0025	5.50	2.33
	0.005	5.33	2.50
	0.0075	4.00	3.83
	0.01	4.83	3.00
	0.025	≤ 2.50	≥ 5.33
3	Hard water	7.17	
	0.0025	5.50	1.67
	0.005	4.00	3.17
	0.0075	3.83	3.33
	0.01	≤ 2.67	≥ 4.50
	0.025	≤ 2.50	≥ 4.67

Table S10. NDV on Douglas fir wood with peracetic acid at 10 °C and 60 min exposure time.

Experiment	Concentration in % (v/v)	Titre in $log_{10}TCID_{50}/mL$	Reduction in $log_{10}TCID_{50}/mL$
1	Hard water	7.33	
	0.0075	4.33	3.00
	0.01	≤ 3.50	≥ 3.83
	0.025	≤ 2.67	≥ 4.67
	0.05	≤ 3.17	≥ 4.17
	0.075	≤ 2.50	≥ 4.83
2	Hard water	7.33	
	0.0075	3.50	3.83
	0.01	≤ 2.50	≥ 4.83
	0.025	≤ 2.50	≥ 4.83
	0.05	≤ 2.50	≥ 4.83
	0.075	≤ 2.50	≥ 4.83
3	Hard water	7.17	
	0.005	4.83	2.33
	0.0075	≤ 2.50	≥ 4.67
	0.01	≤ 2.50	≥ 4.67
	0.025	≤ 2.50	≥ 4.67
	0.05	≤ 2.50	≥ 4.67

Table S11. EV-E on spruce wood with formic acid at 10 °C and 60 min exposure time.

Experiment	Concentration in % (v/v)	Titre in $log_{10}TCID_{50}/mL$	Reduction in $log_{10}TCID_{50}/mL$
1	Hard water	7.00	
	0.25	6.83	0.17
	0.5	6.00	1.00
	0.75	3.83	3.17
	1	≤ 3.50	≥ 3.50
	1.5	≤ 2.50	≥ 4.50
2	Hard water	7.50	
	0.25	7.33	0.17
	0.5	6.50	1.00
	0.75	4.67	2.83
	1	5.50	2.00
	1.5	≤ 3.17	≥ 4.33
3	Hard water	8.17	
	0.25	7.17	1.00
	0.5	7.33	0.83
	0.75	5.17	3.00
	1	3.67	4.50
	1.5	≤ 2.50	≥ 5.67

Table S12. EV-E on pine wood with formic acid at 10 °C and 60 min exposure time.

Experiment	Concentration in % (v/v)	Titre in $log_{10}TCID_{50}/mL$	Reduction in $log_{10}TCID_{50}/mL$
1	Hard water	7.67	
	0.25	6.50	1.17
	0.5	5.50	2.17
	0.75	5.83	1.83
	1	≤ 3.33	≥ 4.33
	1.5	≤ 2.50	≥ 5.17
2	Hard water	7.50	
	0.25	7.17	0.33
	0.5	5.50	2.00
	0.75	4.83	2.67
	1	≤ 2.67	≥ 4.83
	1.5	≤ 2.50	≥ 5.00
3	Hard water	7.67	
	0.25	7.50	0.17
	0.5	5.50	2.17
	0.75	≤ 2.83	≥ 4.83
	1	≤ 2.83	≥ 4.83
	1.5	≤ 2.50	≥ 5.17

Table S13. EV-E on poplar wood with formic acid at 10 °C and 60 min exposure time.

Experiment	Concentration in % (v/v)	Titre in $log_{10}TCID_{50}/mL$	Reduction in $log_{10}TCID_{50}/mL$
1	Hard water	6.83	
	0.25	6.67	0.17
	0.5	5.50	1.33
	0.75	4.17	2.67
	1	≤ 3.00	≥ 3.83
	1.5	≤ 2.50	≥ 4.33
2	Hard water	7.50	
	0.25	7.00	0.50
	0.5	5.67	1.83
	0.75	4.50	3.00
	1	≤ 2.50	≥ 5.00
	1.5	≤ 2.67	≥ 4.83
3	Hard water	7.17	
	0.25	7.00	0.17
	0.5	5.17	2.00
	0.75	4.17	3.00
	1	≤ 2.50	≥ 4.67
	1.5	≤ 2.50	≥ 4.67

Table S14. EV-E on beech wood with formic acid at 10 °C and 60 min exposure time.

Experiment	Concentration in % (v/v)	Titre in $log_{10}TCID_{50}/mL$	Reduction in $log_{10}TCID_{50}/mL$
1	Hard water	7.83	
	0.25	7.17	0.67
	0.5	6.00	1.83
	0.75	5.17	2.67
	1	≤ 3.00	≥ 4.83
	1.5	≤ 2.50	≥ 5.33
2	Hard water	8.00	
	0.25	7.33	0.67
	0.5	6.17	1.83
	0.75	4.50	3.50
	1	≤ 2.83	≥ 5.17
	1.5	≤ 2.50	≥ 5.50
3	Hard water	7.83	
	0.25	7.00	0.83
	0.5	6.17	1.67
	0.75	3.83	4.00
	1	≤ 3.17	≥ 4.67
	1.5	≤ 2.83	≥ 5.00

Table S15. EV-E on Douglas fir wood with formic acid at 10 °C and 60 min exposure time.

Experiment	Concentration in % (v/v)	Titre in $log_{10}TCID_{50}/mL$	Reduction in $log_{10}TCID_{50}/mL$
1	Hard water	7.67	
	0.25	7.17	0.50
	0.5	4.17	3.50
	0.75	≤ 2.50	≥ 5.17
	1	≤ 2.50	≥ 5.17
	1.5	≤ 2.50	≥ 5.17
2	Hard water	7.67	
	0.25	6.67	1.00
	0.5	4.50	3.17
	0.75	3.67	4.00
	1	≤ 2.50	≥ 5.17
	1.5	≤ 2.50	≥ 5.17
3	Hard water	7.83	
	0.25	6.50	1.33
	0.5	4.17	3.67
	0.75	≤ 2.67	≥ 5.17
	1	≤ 2.67	≥ 5.17
	1.5	≤ 2.50	≥ 5.33

Table S16. NDV on spruce wood with formic acid at 10 °C and 60 min exposure time.

Experiment	Concentration in % (v/v)	Titre in $log_{10}TCID_{50}/mL$	Reduction in $log_{10}TCID_{50}/mL$
1	Hard water	6.50	
	0.25	≤ 3.00	≥ 3.50
	0.5	≤ 2.50	≥ 4.00
	0.75	≤ 2.50	≥ 4.00
	1	≤ 2.50	≥ 4.00
	1.5	≤ 2.50	≥ 4.00
2	Hard water	6.67	
	0.075	4.83	1.83
	0.1	4.00	2.67
	0.25	≤ 2.50	≥ 4.17
	0.5	≤ 2.50	≥ 4.17
	0.75	≤ 2.50	≥ 4.17
3	Hard water	6.67	
	0.075	5.17	1.50
	0.1	4.83	1.83
	0.25	≤ 3.17	≥ 3.50
	0.5	≤ 2.50	≥ 4.17
	0.75	≤ 2.50	≥ 4.17

Table S17. NDV on pine wood with formic acid at 10 °C and 60 min exposure time.

Experiment	Concentration in % (v/v)	Titre in $log_{10}TCID_{50}$/mL	Reduction in $log_{10}TCID_{50}$/mL
1	Hard water	6.00	
	0.025	4.00	2.00
	0.05	4.33	1.67
	0.075	3.67	2.33
	0.1	≤ 2.17	≥ 3.83
	0.25	≤ 1.50	≥ 4.50
2	Hard water	6.00	
	0.025	4.83	1.17
	0.05	4.67	1.33
	0.075	4.17	1.83
	0.1	2.67	3.33
	0.25	≤ 1.50	≥ 4.50
3	Hard water	6.17	
	0.025	4.67	1.50
	0.05	3.83	2.33
	0.075	≤ 2.67	≥ 3.50
	0.1	3.67	2.50
	0.25	≤ 1.50	≥ 4.67

Table S18. NDV on poplar wood with formic acid at 10 °C and 60 min exposure time.

Experiment	Concentration in % (v/v)	Titre in $log_{10}TCID_{50}$/mL	Reduction in $log_{10}TCID_{50}$/mL
1	Hard water	6.83	
	0.75	≤ 3.00	≥ 3.83
	1	≤ 2.83	≥ 4.00
	1.5	≤ 2.50	≥ 4.33
	2	≤ 2.50	≥ 4.33
	2.5	≤ 2.50	≥ 4.33
2	Hard water	6.67	
	0.25	4.67	2.00
	0.5	3.83	2.83
	0.75	≤ 3.17	≥ 3.50
	1	≤ 2.50	≥ 4.17
	1.5	≤ 2.50	≥ 4.17
3	Hard water	7.17	
	0.25	4.17	3.00
	0.5	4.00	3.17
	0.75	3.67	3.50
	1	≤ 3.17	≥ 4.00
	1.5	≤ 2.50	≥ 4.67

Table S19. NDV on beech wood with formic acid at 10 °C and 60 min exposure time.

Experiment	Concentration in % (v/v)	Titre in $log_{10}TCID_{50}/mL$	Reduction in $log_{10}TCID_{50}/mL$
1	Hard water	7.50	
	0.25	5.00	2.50
	0.5	4.67	2.83
	0.75	3.83	3.67
	1	4.17	3.33
	1.5	3.50	4.00
2	Hard water	7.50	
	0.5	4.83	2.67
	0.75	4.83	2.67
	1	3.67	3.83
	1.5	≤ 3.33	≥ 4.17
	2	≤ 2.50	≥ 5.00
3	Hard water	7.33	
	0.5	4.83	2.50
	0.75	4.17	3.17
	1	3.67	3.67
	1.5	4.00	3.33
	2	≤ 2.50	≥ 4.83

Table S20. NDV on Douglas fir wood with formic acid at 10 °C and 60 min exposure time.

Experiment	Concentration in % (v/v)	Titre in $log_{10}TCID_{50}/mL$	Reduction in $log_{10}TCID_{50}/mL$
1	Hard water	7.00	
	0.05	5.50	1.50
	0.075	5.33	1.67
	0.1	4.83	2.17
	0.25	≤ 3.00	≥ 4.00
	0.5	≤ 2.67	≥ 4.33
2	Hard water	7.50	
	0.075	6.67	0.83
	0.1	4.83	2.67
	0.25	4.17	3.33
	0.5	4.83	2.67
	0.75	≤ 2.50	≥ 5.00
3	Hard water	7.33	
	0.075	6.67	0.67
	0.1	6.33	1.00
	0.25	4.83	2.50
	0.5	≤ 2.67	≥ 4.67
	0.75	≤ 2.50	≥ 4.83

Table S21. EV-E on spruce wood with glutaraldehyde at 10 °C and 60 min exposure time.

Experiment	Concentration in % (v/v)	Titre in $log_{10}TCID_{50}/mL$	Reduction in $log_{10}TCID_{50}/mL$
1	Hard water	7.50	
	1	5.67	1.83
	2.5	6.00	1.50
	5	≤ 5.00	≥ 2.50
	7.5	≤ 5.00	≥ 2.50
	10	≤ 5.17	≥ 2.33
2	Hard water	7.50	
	1	6.17	1.33
	2.5	≤ 5.33	≥ 2.17
	5	≤ 4.83	≥ 2.67
	7.5	≤ 4.67	≥ 2.83
	10	≤ 4.67	≥ 2.83
3	Hard water	8.00	
	1	6.00	2.00
	2.5	≤ 5.00	≥ 3.00
	5	≤ 4.67	≥ 3.33
	7.5	≤ 4.83	≥ 3.17
	10	≤ 4.50	≥ 3.50

Table S22. EV-E on pine wood with glutaraldehyde at 10 °C and 60 min exposure time.

Experiment	Concentration in % (v/v)	Titre in $log_{10}TCID_{50}/mL$	Reduction in $log_{10}TCID_{50}/mL$
1	Hard water	7.33	
	1	6.67	0.67
	2.5	≤ 5.50	≥ 1.83
	5	≤ 4.67	≥ 2.67
	7.5	≤ 4.83	≥ 2.50
	10	≤ 4.50	≥ 2.83
2	Hard water	7.67	
	1	6.17	1.50
	2.5	≤ 5.67	≥ 2.00
	5	≤ 5.00	≥ 2.67
	7.5	≤ 5.17	≥ 2.50
	10	≤ 4.50	≥ 3.17
3	Hard water	7.33	
	1	6.50	0.83
	2.5	5.50	1.83
	5	≤ 5.17	≥ 2.17
	7.5	≤ 5.00	≥ 2.33
	10	≤ 5.33	≥ 2.00

Table S23. EV-E on poplar wood with glutaraldehyde at 10 °C and 60 min exposure time.

Experiment	Concentration in % (v/v)	Titre in $log_{10}TCID_{50}$/mL	Reduction in $log_{10}TCID_{50}$/mL
1	Hard water	7.00	
	1	6.50	0.50
	2.5	5.67	1.33
	5	5.50	1.50
	7.5	≤ 4.50	≥ 2.50
	10	≤ 5.33	≥ 1.67
2	Hard water	7.50	
	1	6.50	1.00
	2.5	6.00	1.50
	5	≤ 4.50	≥ 3.00
	7.5	≤ 5.17	≥ 2.33
	10	≤ 5.00	≥ 2.50
3	Hard water	7.33	
	1	6.50	0.83
	2.5	6.00	1.33
	5	≤ 4.50	≥ 2.83
	7.5	5.50	1.83
	10	≤ 5.50	≥ 1.83

Table S24. EV-E on beech wood with glutaraldehyde at 10 °C and 60 min exposure time.

Experiment	Concentration in % (v/v)	Titre in $log_{10}TCID_{50}$/mL	Reduction in $log_{10}TCID_{50}$/mL
1	Hard water	8.17	
	1	6.50	1.67
	2.5	5.83	2.33
	5	5.67	2.50
	7.5	5.67	2.50
	10	≤ 5.33	≥ 2.83
2	Hard water	8.00	
	1	6.00	2.00
	2.5	5.50	2.50
	5	≤ 5.33	≥ 2.67
	7.5	≤ 4.83	≥ 3.17
	10	≤ 5.17	≥ 2.83
3	Hard water	7.67	
	1	6.50	1.17
	2.5	6.17	1.50
	5	≤ 5.00	≥ 2.67
	7.5	≤ 5.17	≥ 2.50
	10	≤ 4.83	≥ 2.83

Table S25. EV-E on Douglas fir wood with glutaraldehyde at 10 °C and 60 min exposure time.

Experiment	Concentration in % (v/v)	Titre in $log_{10}TCID_{50}/mL$	Reduction in $log_{10}TCID_{50}/mL$
1	Hard water	6.50	
	1	≤ 5.33	≥ 1.17
	2.5	≤ 5.17	≥ 1.33
	5	≤ 4.83	≥ 1.67
	7.5	≤ 4.67	≥ 1.83
	10	≤ 4.50	≥ 2.00
2	Hard water	7.17	
	1	≤ 5.33	≥ 1.83
	2.5	≤ 5.17	≥ 2.00
	5	≤ 5.00	≥ 2.17
	7.5	≤ 4.50	≥ 2.67
	10	≤ 4.67	≥ 2.50
3	Hard water	7.33	
	1	6.17	1.17
	2.5	5.67	1.67
	5	≤ 4.50	≥ 2.83
	7.5	≤ 5.33	≥ 2.00
	10	≤ 4.67	≥ 2.67

Table S26. NDV on spruce wood with glutaraldehyde at 10 °C and 60 min exposure time.

Experiment	Concentration in % (v/v)	Titre in $log_{10}TCID_{50}/mL$	Reduction in $log_{10}TCID_{50}/mL$
1	Hard water	6.50	
	0.25	≤ 3.00	≥ 3.50
	0.5	≤ 2.83	≥ 3.67
	0.75	≤ 2.50	≥ 4.00
	1	≤ 2.50	≥ 4.00
	1.5	≤ 2.50	≥ 4.00
2	Hard water	6.50	
	0.1	3.67	2.83
	0.25	≤ 3.17	≥ 3.33
	0.5	≤ 2.50	≥ 4.00
	0.75	≤ 2.50	≥ 4.00
	1	≤ 2.50	≥ 4.00
3	Hard water	6.50	
	0.1	3.50	3.00
	0.25	≤ 3.17	≥ 3.33
	0.5	≤ 2.67	≥ 3.83
	0.75	≤ 2.50	≥ 4.00
	1	≤ 2.50	≥ 4.00

Table S27. NDV on pine wood with glutaraldehyde at 10 °C and 60 min exposure time.

Experiment	Concentration in % (v/v)	Titre in $log_{10}TCID_{50}/mL$	Reduction in $log_{10}TCID_{50}/mL$
1	Hard water	6.83	
	0.075	3.33	3.50
	0.1	≤ 1.50	≥ 5.33
	0.25	≤ 1.50	≥ 5.33
	0.5	≤ 1.50	≥ 5.33
	0.75	≤ 1.50	≥ 5.33
2	Hard water	5.67	
	0.025	3.33	2.33
	0.05	2.67	3.00
	0.075	≤ 1.67	≥ 4.00
	0.1	≤ 1.50	≥ 4.17
	0.25	≤ 1.50	≥ 4.17
3	Hard water	6.17	
	0.025	3.50	2.67
	0.05	2.50	3.67
	0.075	≤ 2.17	≥ 4.00
	0.1	≤ 1.50	≥ 4.67
	0.25	≤ 1.50	≥ 4.67

Table S28. NDV on poplar wood with glutaraldehyde at 10 °C and 60 min exposure time.

Experiment	Concentration in % (v/v)	Titre in $log_{10}TCID_{50}/mL$	Reduction in $log_{10}TCID_{50}/mL$
1	Hard water	6.83	
	0.25	3.67	3.17
	0.5	≤ 3.17	≥ 3.67
	0.75	4.17	2.67
	1	≤ 3.33	≥ 3.50
	1.5	≤ 2.67	≥ 4.17
2	Hard water	6.83	
	1	≤ 3.00	≥ 3.83
	1.5	≤ 2.50	≥ 4.33
	2	≤ 2.50	≥ 4.33
	2.5	≤ 2.50	≥ 4.33
	3	≤ 2.50	≥ 4.33
3	Hard water	6.67	
	1	≤ 2.83	≥ 3.83
	1.5	≤ 2.50	≥ 4.17
	2	≤ 2.50	≥ 4.17
	2.5	≤ 2.50	≥ 4.17
	3	≤ 2.50	≥ 4.17

Table S29. NDV on beech wood with glutaraldehyde at 10 °C and 60 min exposure time.

Experiment	Concentration in % (v/v)	Titre in $log_{10}TCID_{50}/mL$	Reduction in $log_{10}TCID_{50}/mL$
1	Hard water	7.50	
	0.75	3.67	3.83
	1	3.83	3.67
	1.5	≤ 2.50	≥ 5.00
	2	≤ 2.50	≥ 5.00
	2.5	≤ 2.50	≥ 5.00
2	Hard water	7.50	
	0.5	3.83	3.67
	0.75	3.67	3.83
	1	4.00	3.50
	1.5	≤ 2.67	≥ 4.83
	2	≤ 2.50	≥ 5.00
3	Hard water	7.33	
	0.5	4.50	2.83
	0.75	≤ 3.50	≥ 3.83
	1	≤ 2.67	≥ 4.67
	1.5	≤ 2.50	≥ 4.83
	2	≤ 2.50	≥ 4.83

Table S30. NDV on Douglas fir wood with glutaraldehyde at 10 °C and 60 min exposure time.

Experiment	Concentration in % (v/v)	Titre in $log_{10}TCID_{50}/mL$	Reduction in $log_{10}TCID_{50}/mL$
1	Hard water	7.83	
	0.25	4.00	3.83
	0.5	≤ 3.17	≥ 4.67
	0.75	≤ 2.50	≥ 5.33
	1	≤ 3.33	≥ 4.50
	1.5	≤ 2.50	≥ 5.33
2	Hard water	7.67	
	0.075	5.00	2.67
	0.1	4.67	3.00
	0.25	5.33	2.33
	0.5	≤ 2.67	≥ 5.00
	0.75	≤ 2.50	≥ 5.17
3	Hard water	7.50	
	0.075	4.83	2.67
	0.1	4.83	2.67
	0.25	4.17	3.33
	0.5	≤ 3.83	≥ 3.67
	0.75	≤ 2.67	≥ 4.83

Table S31. EV-E on spruce wood with peracetic acid at −10 °C and 60 min exposure time.

Experiment	Concentration in % (v/v)	Titre in $log_{10}TCID_{50}$/mL	Reduction in $log_{10}TCID_{50}$/mL
1	Hard water	8.33	
	0.1	5.33	3.00
	0.25	≤ 3.50	≥ 4.83
	0.5	≤ 2.83	≥ 5.50
	0.75	≤ 2.50	≥ 5.83
	1	≤ 2.50	≥ 5.83
2	Hard water	8.00	
	0.05	5.83	2.17
	0.075	4.83	3.17
	0.1	5.00	3.00
	0.25	≤ 3.17	≥ 4.83
	0.5	≤ 2.67	≥ 5.33
3	Hard water	8.17	
	0.05	5.67	2.50
	0.075	5.33	2.83
	0.1	5.17	3.00
	0.25	≤ 2.83	≥ 5.33
	0.5	≤ 2.50	≥ 5.67

Table S32. EV-E on pine wood with peracetic acid at −10 °C and 60 min exposure time.

Experiment	Concentration in % (v/v)	Titre in $log_{10}TCID_{50}$/mL	Reduction in $log_{10}TCID_{50}$/mL
1	Hard water	7.67	
	0.05	5.50	2.17
	0.075	5.17	2.50
	0.1	5.17	2.50
	0.25	4.50	3.17
	0.5	≤ 2.67	≥ 5.00
2	Hard water	8.17	
	0.05	5.83	2.33
	0.075	5.17	3.00
	0.1	6.00	2.17
	0.25	4.83	3.33
	0.5	≤ 4.00	≥ 4.17
3	Hard water	7.67	
	0.075	5.67	2.00
	0.1	5.67	2.00
	0.25	4.33	3.33
	0.5	3.50	4.17
	0.75	≤ 3.50	≥ 4.17

Table S33. EV-E on poplar wood with peracetic acid at −10 °C and 60 min exposure time.

Experiment	Concentration in % (v/v)	Titre in $log_{10}TCID_{50}/mL$	Reduction in $log_{10}TCID_{50}/mL$
1	Hard water	7.83	
	0.05	5.17	2.67
	0.075	5.33	2.50
	0.1	4.17	3.67
	0.25	≤ 2.83	≥ 5.00
	0.5	≤ 3.17	≥ 4.67
2	Hard water	8.17	
	0.05	5.17	3.00
	0.075	4.50	3.67
	0.1	5.67	2.50
	0.25	≤ 3.33	≥ 4.83
	0.5	≤ 2.50	≥ 5.67
3	Hard water	7.50	
	0.05	5.17	2.33
	0.075	4.50	3.00
	0.1	4.00	3.50
	0.25	≤ 2.67	≥ 4.83
	0.5	≤ 2.50	≥ 5.00

Table S34. EV-E on beech wood with peracetic acid at −10 °C and 60 min exposure time.

Experiment	Concentration in % (v/v)	Titre in $log_{10}TCID_{50}/mL$	Reduction in $log_{10}TCID_{50}/mL$
1	Hard water	7.83	
	0.075	5.33	2.50
	0.1	5.33	2.50
	0.25	4.00	3.83
	0.5	≤ 3.17	≥ 4.67
	0.75	≤ 2.50	≥ 5.33
2	Hard water	7.83	
	0.075	5.67	2.17
	0.1	5.33	2.50
	0.25	4.17	3.67
	0.5	4.67	3.17
	0.75	≤ 2.50	≥ 5.33
3	Hard water	7.83	
	0.075	5.17	2.67
	0.1	5.17	2.67
	0.25	4.00	3.83
	0.5	≤ 2.67	≥ 5.17
	0.75	≤ 2.50	≥ 5.33

Table S35. EV-E on Douglas fir wood with peracetic acid at −10 °C and 60 min exposure time.

Experiment	Concentration in % (v/v)	Titre in $log_{10}TCID_{50}/mL$	Reduction in $log_{10}TCID_{50}/mL$
1	Hard water	7.67	
	0.075	5.83	1.83
	0.1	4.67	3.00
	0.25	4.50	3.17
	0.5	≤ 2.50	≥ 5.17
	0.75	≤ 2.50	≥ 5.17
2	Hard water	7.83	
	0.075	6.50	1.33
	0.1	5.50	2.33
	0.25	5.50	2.33
	0.5	≤ 3.33	≥ 4.50
	0.75	≤ 3.50	≥ 4.33
3	Hard water	7.17	
	0.075	5.67	1.50
	0.1	5.50	1.67
	0.25	4.00	3.17
	0.5	≤ 2.50	≥ 4.67
	0.75	≤ 2.67	≥ 4.50

Table S36. EV-E on spruce wood with formic acid at −10 °C and 60 min exposure time.

Experiment	Concentration in % (v/v)	Titre in $log_{10}TCID_{50}/mL$	Reduction in $log_{10}TCID_{50}/mL$
1	Hard water	7.00	
	1	5.83	1.17
	1.5	4.67	2.33
	2	≤ 3.33	≥ 3.67
	2.5	3.83	3.17
	3	≤ 2.50	≥ 4.50
2	Hard water	7.67	
	1.5	5.17	2.50
	2	4.17	3.50
	2.5	4.17	3.50
	3	≤ 2.67	≥ 5.00
	3.5	≤ 2.50	≥ 5.17
3	Hard water	7.50	
	1.5	3.83	3.67
	2	4.00	3.50
	2.5	≤ 2.67	≥ 4.83
	3	≤ 2.50	≥ 5.00
	3.5	≤ 2.50	≥ 5.00

Table S37. EV-E on pine wood with formic acid at −10 °C and 60 min exposure time.

Experiment	Concentration in % (v/v)	Titre in $log_{10}TCID_{50}/mL$	Reduction in $log_{10}TCID_{50}/mL$
1	Hard water	7.50	
	2	5.00	2.50
	3	3.83	3.67
	4	3.83	3.67
	5	≤ 2.50	≥ 5.00
	6	≤ 2.50	≥ 5.00
2	Hard water	7.67	
	2	5.67	2.00
	3	4.83	2.83
	4	4.17	3.50
	5	≤ 2.50	≥ 5.17
	6	≤ 3.67	≥ 4.00
3	Hard water	7.50	
	2	5.00	2.50
	3	4.00	3.50
	4	4.17	3.33
	5	≤ 3.17	≥ 4.33
	6	≤ 2.50	≥ 5.00

Table S38. EV-E on poplar wood with formic acid at −10 °C and 60 min exposure time.

Experiment	Concentration in % (v/v)	Titre in $log_{10}TCID_{50}/mL$	Reduction in $log_{10}TCID_{50}/mL$
1	Hard water	7.33	
	1	5.67	1.67
	1.5	5.00	2.33
	2	4.50	2.83
	2.5	≤ 2.67	≥ 4.67
	3	≤ 3.17	≥ 4.17
2	Hard water	7.17	
	1.5	5.83	1.33
	2	4.67	2.50
	2.5	≤ 3.33	≥ 3.83
	3	≤ 2.50	≥ 4.67
	3.5	≤ 2.50	≥ 4.67
3	Hard water	7.33	
	2	5.00	2.33
	2.5	4.17	3.17
	3	≤ 2.67	≥ 4.67
	3.5	≤ 2.50	≥ 4.83
	4	≤ 2.50	≥ 4.83

Table S39. EV-E on beech wood with formic acid at −10 °C and 60 min exposure time.

Experiment	Concentration in % (v/v)	Titre in $\log_{10}TCID_{50}/mL$	Reduction in $\log_{10}TCID_{50}/mL$
1	Hard water	8.00	
	2	4.83	3.17
	2.5	3.83	4.17
	3	≤ 3.17	≥ 4.83
	3.5	≤ 2.50	≥ 5.50
	4	≤ 2.83	≥ 5.17
2	Hard water	7.50	
	1.5	5.00	2.50
	2	4.83	2.67
	2.5	4.17	3.33
	3	≤ 3.33	≥ 4.17
	3.5	≤ 2.83	≥ 4.67
3	Hard water	7.67	
	1.5	5.33	2.33
	2	4.67	3.00
	2.5	≤ 3.33	≥ 4.33
	3	≤ 2.50	≥ 5.17
	3.5	≤ 3.33	≥ 4.33

Table S40. EV-E on Douglas fir wood with formic acid at −10 °C and 60 min exposure time.

Experiment	Concentration in % (v/v)	Titre in $\log_{10}TCID_{50}/mL$	Reduction in $\log_{10}TCID_{50}/mL$
1	Hard water	7.17	
	2	4.33	2.83
	2.5	3.50	3.67
	3	≤ 3.33	≥ 3.83
	3.5	≤ 2.50	≥ 4.67
	4	≤ 2.50	≥ 4.67
2	Hard water	6.83	
	2	4.83	2.00
	2.5	4.00	2.83
	3	≤ 2.50	≥ 4.33
	3.5	≤ 2.50	≥ 4.33
	4	≤ 2.50	≥ 4.33
3	Hard water	7.17	
	2	5.50	1.67
	2.5	4.83	2.33
	3	≤ 3.33	≥ 3.83
	3.5	≤ 3.83	≥ 3.33
	4	≤ 3.17	≥ 4.00
